# Nomogram-based model to predict prognosis in anisometropic amblyopia

**DOI:** 10.3389/fmed.2025.1612845

**Published:** 2025-10-13

**Authors:** Wenyan Xu, Xiaoman Li, Lizhong Wang, Xiyan Xiang, Yuejia Peng, Hongyi Li, Xuewen Ding, Jianing Zhang, Xiaoyue Hu, Jie Chen, Wuhe Chen

**Affiliations:** National Clinical Research Center for Ocular Diseases, Eye Hospital, Wenzhou Medical University, Wenzhou, Zhejiang, China

**Keywords:** anisometropic amblyopia, prognosis, nomograms, predictive model, best corrected visual acuity

## Abstract

**Purpose:**

This study aimed to identify predictive factors and develop an effective nomogram to estimate the prognosis of anisometropic amblyopia.

**Methods:**

We retrospectively analyzed 2,897 patients aged 3–18 years diagnosed with anisometropic amblyopia, with at least 12 months of follow-up. The cure criterion was a best corrected visual acuity (BCVA) of 0.1 LogMAR or better in the amblyopic eye, with less than one line of interocular difference. The potential predictors included 15 baseline clinical characteristics. Participants were randomly assigned (7:3) to the training and validation sets. A nomogram to predict the prognosis of amblyopia was computed using a logistic regression model with least absolute shrinkage and a selection operator. Model performance was assessed by discrimination (area under the curve [AUC]) and calibration (calibration plots).

**Results:**

This study included 2,897 patients, of whom 35.2% met the criteria for cured amblyopia. The training and validation sets comprised 2,040 and 857 participants, respectively. The predictors included in the nomogram were baseline age (AUC: 0.703 [95% CI 0.679–0.727]), difference in interocular BCVA (AUC: 0.688 [95% CI 0.664–0.711]), and spherical equivalence (SE) (AUC: 0.653 [95% CI 0.629–0.678]). The calibration curves of the nomogram showed good agreement between the predicted and observed probabilities, with an AUC of 0.783 (95% CI 0.763–0.803) in the training set and 0.782 (95% CI 0.750–0.814) in the validation set.

**Conclusion:**

The nomogram, incorporating baseline age, differences in interocular BCVA, and SE, provides individualized prognostic predictions for anisometropic amblyopia treatment, aiding clinicians in personalized treatment planning and better resource allocation. Furthermore, the nomogram could enhance shared decision-making with parents by providing objective prognostic data, thus improving treatment compliance.

## 1 Introduction

Amblyopia is a condition in which the best corrected visual acuity (BCVA) of one or both eyes falls below the age-appropriate standard during the visual development period due to factors such as unilateral strabismus, anisometropia, high refractive error, or form deprivation. A meta-analysis of 97 studies reported a global amblyopia prevalence of 1.36% (95% CI 1.27–1.46%) (1). During visual development, any factor that prevents both eyes from receiving the same information can result in unilateral amblyopia. Anisometropia is the most important cause, with studies reporting that 1/3 of amblyopia is caused by anisometropia, and some reports suggesting 50%, with this proportion exceeding half of cases in China (2–5).

Current common treatments for anisometropic amblyopia include refractive correction and blocking visual input in the healthy eye using patching or optical or pharmaceutical penalization (6, 7). Previous studies have indicated that multiple parameters may affect the therapeutic efficacy of amblyopia (8–14), making personalized treatment for amblyopia particularly important (15, 16). However, a targeted method for prognostic evaluation in the treatment of anisometropic amblyopia is lacking, which hinders the implementation of personalized management strategies in these patients. Therefore, predicting the prognosis of amblyopia treatment based on baseline characteristics will likely facilitate the establishment of personalized treatment plans, optimize resource allocation, and promote therapeutic consensus with parents through visualized outcome data, collectively enhancing treatment compliance and health-economic outcomes in amblyopia management (16).

Nomogram is a graphical tool that translates a multivariable statistical model into an easy-to-use format, allowing clinicians to estimate individualized probabilities of treatment outcomes. Nomograms are widely used in medicine to establish models for predicting disease prognosis (17–19), assisting in tailoring personalized treatment plans and guiding clinical decisions. In ophthalmology, studies have examined areas such as predicting the efficacy of orthokeratology and the risk of second surgery in patients with concomitant esotropia (20–24). This study retrospectively analyzed a large dataset of electronic medical records from patients with anisometropic amblyopia to identify predictive factors for amblyopia prognosis, evaluate their discriminative ability, and establish a predictive model using a nomogram. In the final part of this article, we will present this graphical nomogram, which translates baseline clinical factors into an individualized prediction of treatment outcome.

## 2 Materials and methods

### 2.1 Study population

This retrospective study included patients diagnosed with anisometropic amblyopia at the Eye Hospital of Wenzhou Medical University between February 2003 and September 2023. The inclusion criteria were as follows:

(1) A BCVA difference of at least two lines between the eyes due to anisometropia.
(2) Anisohyperopia of 1.50 D or greater between the amblyopic eye and the healthy eye, or anisoastigmatism of 2.00 D or greater, or anisomyopia of 3.00 D or greater.
(3) Age at initial diagnosis between 3 and 18 years.
(4) At least 12 months between the initial and final follow-up record.

Patients with neurological or ocular diseases, such as cataracts, glaucoma, fundus disease, and nystagmus, were excluded. This study adhered to the Declaration of Helsinki and was approved by the Ethics Committee of the Eye Hospital of Wenzhou Medical University (Approval No. 2023-112-K-89). As this was a retrospective study, the requirement for informed consent was waived by the Ethics Committee.

### 2.2 Measurements and definitions

Refractive error was measured using an autorefractor, followed by subjective refraction, with the initial refraction performed under cycloplegia. For patients under 10 years of age, refraction was conducted using retinoscopy combined with the lens-insertion method; in contrast, those aged 10 years or older underwent refraction using a phoropter. Visual acuity assessment is conducted in an environment with uniform and constant brightness, using a standard logarithmic visual acuity chart. During the test, the two eyes are examined separately, following the order of right eye first and then left eye. During the examination, the contralateral eye must be completely covered with an occluder that thoroughly blocks the entry of light. The subject is required to identify the optotypes row by row from the top to the bottom of the visual acuity chart until they are unable to accurately recognize all the optotypes in a certain row. At this point, the visual acuity value corresponding to the lowest row of optotypes that the subject can fully recognize is recorded as the base value. If the subject can still recognize some optotypes in the row immediately below this fully recognizable row, the final visual acuity value is calculated by adding “the number of recognized optotypes in the partially recognizable row × 0.02 LogMAR” to the base visual acuity value. All visual acuity results are converted to the LogMAR chart for analysis. After the initial diagnosis of anisometropic amblyopia, all patients received full spectacle correction and treatment by occluding the sound eye through patching. The duration of patching was determined by the severity of amblyopia and the patient’s response to treatment. Regular follow-up visits were required throughout the treatment period.

The cured group was defined as patients whose final BCVA was 0.1 LogMAR or better, with the BCVA of the amblyopic eye within one line of the unaffected eye at the final follow-up. Amblyopia was considered uncured if these criteria were not met.

### 2.3 Predictive variables and sample size

This study collected 16 independent baseline variables routinely recorded in the electronic medical record system, including the difference in interocular spherical equivalence (SE), spherical power (SPH), cylindrical power (CYL), and BCVA; SE, SPH, CYL, and BCVA of the amblyopic eye; the laterality of the amblyopic eye (left or right); the type of refractive error in the amblyopic eye (hyperopia or myopia); whether the amblyopic eye has with-the-rule astigmatism (1–15° or 165–180°); whether the amblyopic eye has against-the-rule astigmatism (75–105°); whether the amblyopic eye has oblique astigmatism (16–74° or 106–164°); whether strabismus is combined; gender; and age.

According to Harrell’s guideline (also known as the 10-EPV criterion, meaning the number of events should be at least 10 times the number of independent variables) for multivariate logistic regression (25, 26), the sample size for the cured group in this study should be at least 120 cases. In previous studies, a cure rate of 45.10 to 54.8% was observed in patients with anisometropic amblyopia (12, 27, 28). Using 45% as the estimated cure rate, the required sample size for training in this study was at least 120/45% = 267. Because the training cohort constituted approximately 70% of the total sample size, the total sample should be at least 267/70% = 381 cases, with 114 cases used to validate the model. In this study, 2,897 participants were collected and divided into a training set (*n* = 2,040) and a validation set (*n* = 857) at a 7:3 ratio. Notably, a larger sample size contributes to reducing estimation errors and overfitting risks, thereby enhancing the model’s generalizability.

To validate the statistical power, a DeLong test was performed with the following formula: $n=\frac{{\left(Z_{1-\alpha /2}+Z_{1-\beta }\right)}^{2}\times 2\times {\left(1-\rho \right)}^{2}}{{\left({\mathrm{AUC}}_{1}-{\mathrm{AUC}}_{0}\right)}^{2}}$ . Assuming a target area under the receiver operating characteristic (ROC) curve (AUC) of 0.75 (clinically meaningful discriminative ability) and a null hypothesis of AUC = 0.5. Using a two-sided test with *α* = 0.05 (Z₁₋*α*/₂ = 1.96) and a conservative correlation coefficient (*ρ* = 0.5) between predicted probabilities, the training set sample size (*n* = 2,040) was used to reverse-calculate power. This yielded Z_₁₋*β*_≈14.01 (*β* ≈ 10–44), corresponding to a power (1 − *β*) > 99.9%.

### 2.4 Statistical analyses

Statistical analyses were conducted using SPSS Statistics (version 27.0; IBM Corp., Armonk, NY, USA) and R version 4.2.3 (R Foundation for Statistical Computing), with statistical significance set at *p* < 0.05. Continuous variables are reported as medians (interquartile ranges). The Shapiro–Wilk test was used to examine whether the variables were normally distributed. The Mann–Whitney U or chi-squared test was used to verify the consistency between the training and validation sets.

Predictor selection was performed using LASSO regression and 10-fold cross-validation. LASSO is a machine learning algorithm that prevents model overfitting and enhances its generalization ability. To test multicollinearity, variance inflation factors (VIF) were calculated using linear regression analysis. Subsequently, a multivariable logistic regression model based on forward stepwise selection was constructed to select the most valuable variables (*p* < 0.001). The performance of candidate models was initially compared using the Vuong test and 5-fold cross-validation based on the training set. Model fit was assessed using the Hosmer-Lemeshow test, Akaike information criterion (AIC), and Bayesian information criterion (BIC). The discriminative ability of each variable in the prediction model, as well as the overall predictive accuracy and discrimination of the model, were quantified using the AUC of the ROC curve. Calibration curves were plotted to assess the concordance between nomogram-estimated and actual probabilities. Clinical usefulness and net benefit of the nomogram were evaluated with decision curve analysis (DCA). Additionally, continuous variables in the model were subjected to nonlinear transformation using restricted cubic splines (RCS) with 4 internal knots, and their overall effects (including linear and nonlinear components) and nonlinear effects were evaluated via the Wald test.

## 3 Results

This study included 2,897 participants. The baseline age was 9.67 (6.72, 12.29) years, with a median follow-up duration of 2.83 (1.71, 4.87) years. At the final follow-up, the age was 13.05 (9.73, 16.57) years, and the visual acuity of the amblyopic eye was 0.22 (0.05, 0.50) LogMAR. During the treatment period, the difference in interocular BCVA improved from 0.52 (0.30, 0.80) LogMAR to 0.22 (0.08, 0.50) LogMAR.

The total study population was randomly divided into training (*n* = 2040) and validation (*n* = 857) sets in a 7:3 ratio. There were no significant differences between the training and validation sets in terms of baseline characteristics, follow-up duration, or treatment outcomes (all *p* > 0.05, Table 1).

**Table 1:** Characteristics of the training and validation sets.

Variables	Median (Interquartile range) / Proportion		*Z* / *χ*^2^	*p* value
	Training set (*n* = 2040)	Validation set (*n* = 857)		
Baseline difference in interocular SE (D)	3.88 (2.50, 5.38)	4.00 (2.38, 5.63)	−1.031	0.303
Baseline difference in interocular SPH (D)	4.00 (2.75, 5.69)	4.00 (2.75, 5.75)	−0.725	0.469
Baseline difference in interocular CYL (D)	−0.75 (−1.50, 0.00)	−0.75 (−1.50, 0.00)	−0.221	0.825
Baseline difference in interocular BCVA (LogMAR)	0.52 (0.30, 0.80)	0.52 (0.30, 0.80)	−0.196	0.845
Baseline SE of amblyopic eye (D)	4.00 (2.00, 5.50)	4.00 (1.75, 5.63)	0.125	0.900
Baseline SPH of amblyopic eye (D)	4.50 (2.75, 6.00)	4.50 (2.50, 6.00)	0.227	0.820
Baseline CYL of amblyopic eye (D)	−1.00 (−1.75, −0.50)	−1.00 (−2.00, −0.50)	0.027	0.978
Baseline BCVA of amblyopic eye (LogMAR)	0.52 (0.40, 0.80)	0.52 (0.40, 0.81)	−0.204	0.839
Gender (male / female)*	1,146 / 894	464 / 393	1.011	0.315
Baseline age (years)	9.82 (6.70, 12.23)	9.34 (6.82, 12.32)	−0.285	0.776
Amblyopic eye (right / left) *	795 / 1,245	347/ 510	0.583	0.445
Refractive condition of amblyopic eye (myopia / hyperopia)*	315 / 1725	145 / 712	0.987	0.320
Whether the amblyopic eye is WTR astigmatism (Yes / No)*	1,212 / 828	507 / 350	0.016	0.900
Whether the amblyopic eye is ATR astigmatism (Yes / No)*	86 / 1954	26 / 831	2.268	0.132
Whether the amblyopic eye is oblique astigmatism (Yes / No)*	372 / 1,668	152 / 795	2.134	0.144
Whether strabismus is combined (Yes / No)*	259 / 1781	121 / 776	0.348	0.555
Cure status (cure / uncured)*	727 / 1,313	292 / 565	0.648	0.421

*Proportions were analyzed using the Chi-squared test, and the remaining variables, which are continuous variables, were analyzed using the Mann–Whitney U test.

SE, spherical equivalent; SPH, spherical refractive error; CYL, cylindrical refractive error; BCVA, best-corrected visual acuity; WTR, with-the-rule; ATR, against-the-rule.

### 3.1 Selection of variables for prediction

**Figure 1:**
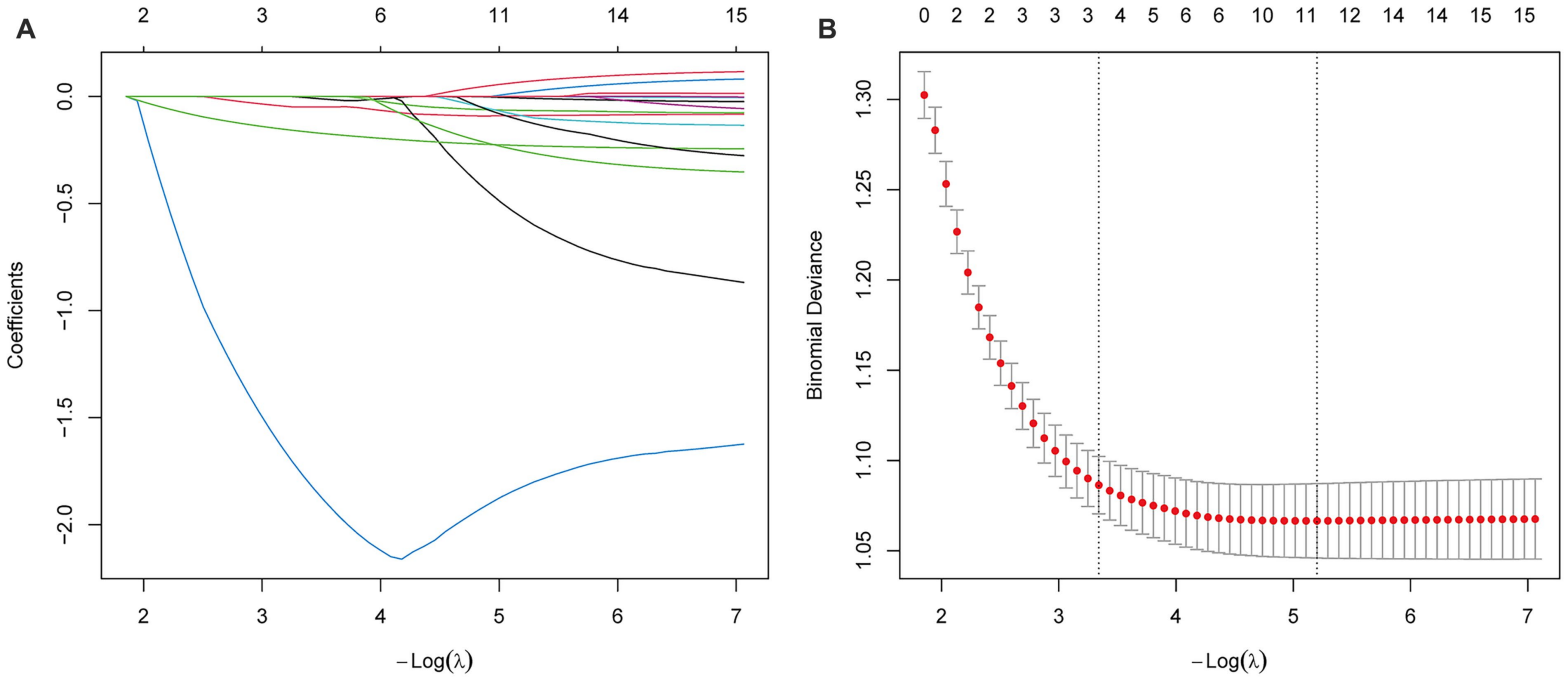
Feature selection by LASSO regression. **(A)** Coefficients of the variables filtered by LASSO showed their importance. **(B)** After 10-fold cross-validation the initial 15 features were reduced to 4 predictors (select the largest regularization parameter within one standard error of the minimum mean squared error).

Baseline characteristics for predicting treatment outcomes were selected from the training set using LASSO regression. A total of 16 variables were involved, and the largest regularization parameter within one standard error of the minimum mean squared error was selected through cross-validation, ultimately retaining four variables: age, difference in interocular SE, SPH, and BCVA (Figure 1).

**Figure 2:**
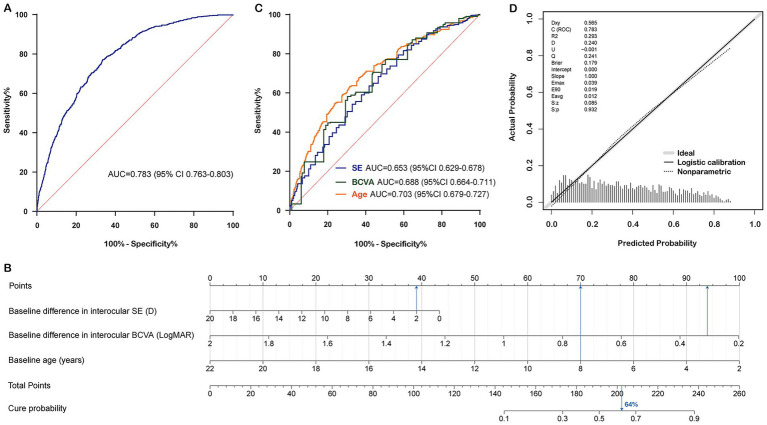
Construction and calibration of the nomogram prediction model in the training set. **(A)** Receiver operating characteristic curve of nomogram for cure status prediction. **(B)** Nomogram for cure status prediction. **(C)** Receiver operating characteristic curves for each predictive factor. **(D)** Calibration curve of nomogram for cure status prediction. Note: The probability calculation formula of the nomogram is: Cure probability = 1/(1 + exp.(−(3.4420 + (−0.1020) × (Baseline difference in interocular SE) + (−2.6117) × (Baseline difference in interocular BCVA) + (−0.2353) × (Baseline age)))). Data from a 10-year-old patient with anisometropic amblyopia is shown as an example. The baseline difference in interocular SE was 2 D, the baseline difference in interocular BCVA was 0.3 LogMAR, and the baseline age was 8 years. By connecting each factor to the “Points” axis (presented in blue), the individual points were calculated, resulting in a total score of approximately 202.5. Drawing a vertical line downwards showed that this patient’s cure probability was 64%. SE, spherical equivalent; BCVA, best corrected visual acuity; AUC, area under the curve.

Owing to the high correlation between the baseline interocular SE and SPH differences (*r* = 0.950, *p* < 0.001, Spearman), two binary logistic regression models were established to avoid potential multicollinearity among the variables. Stepwise forward regression was then performed for further variable selection. Regression Model 1 included baseline age, differences in interocular SE, and BCVA. No collinearity was observed among these variables (VIFs < 5.0). The AUC was 0.783 (95% CI 0.763–0.803) (Figure 2A), AIC was 2175.27, BIC was 2197.76, and the Hosmer-Lemeshow test yielded a chi-squared value of 9.024 with a *p*-value of 0.340. Regression Model 2 included baseline age, differences in interocular SPH, and BCVA. Again, no collinearity was found among these variables (VIFs < 5.0). The AUC was 0.782 (95% CI 0.762–0.802), AIC was 2175.51, BIC was 2197.99, and the Hosmer-Lemeshow test produced a chi-squared value of 10.274 with a *p*-value of 0.246 (Table 2). The Vuong test indicated no significant difference in the fit performance between the two models (*Z* = 0.083, *p* = 0.467). In 5-fold cross-validation, both models had nearly identical root mean squared error (RMSE = 0.424) and mean absolute error (MAE = 0.358). However, the coefficient of determination (R2) for Regression Model 1 was 0.221, and for Regression Model 2, it was 0.219, suggesting that Regression Model 1 fit the data slightly better than Regression Model 2.

**Table 2:** Binary logistic regression models for analyzing factors influencing the cure status of amblyopic eyes.

Variables	Regression model 1 (Hosmer-Lemeshow *χ*^2^ = 9.024, *p* = 0.340)*		Regression model 2 (Hosmer-Lemeshow *χ*^2^ = 10.274, *p* = 0.246)*	
	OR (95% CI)	*p* value	OR (95% CI)	*p* value
Baseline difference in interocular SE (D)	0.903 (0.853–0.956)	<0.001	——	——
Baseline difference in interocular SPH (D)	——	——	0.900 (0.848–0.955)	<0.001
Baseline difference in interocular BCVA (D)	0.073 (0.047–0.115)	<0.001	0.073 (0.047–0.115)	<0.001
Baseline age (years)	0.790 (0.765–0.816)	<0.001	0.792 (0.767–0.819)	<0.001

*Evaluate the model’s goodness of fit using the Hosmer-Lemeshow test. To avoid multicollinearity, the difference in binocular SE and the difference in binocular SPH were each included in two separate binary logistic Abbreviation: regression models for stepwise regression to further screen variables.

SE, spherical equivalent; SPH, spherical power; BCVA, best corrected visual acuity.

### 3.2 Construction and validation of the predictive model

Both regression models demonstrated good fit. Regression Model 1 had a higher AUC than Regression Model 2, along with lower AIC and BIC, and a higher R2, indicating that Regression Model 1 theoretically has a stronger predictive ability and better goodness of fit. Additionally, SE is a comprehensive index that combines SPH and CYL, making it more extensive than SPH for clinical evaluation. This suggests that Regression Model 1 is more suitable than Regression Model 2 for developing the nomogram prediction model (AUC = 0.783 [95% CI 0.763–0.803], AIC = 2175.27, BIC = 2197.76, *R*^2^ = 0.221) (Figure 2B). Younger patients and those with smaller baseline interocular BCVA and SE differences were more likely to achieve recovery. The discriminative ability of each variable in the model was evaluated using ROC curves: the AUC of the baseline interocular SE difference was 0.653 (95% CI 0.629–0.678), the AUC of the interocular BCVA difference was 0.688 (95% CI 0.664–0.711), and the AUC of age was 0.703 (95% CI 0.679–0.727) (Figure 2C).

Furthermore, RCS were used to perform nonlinear transformation on the predictors in Regression Model 1, and Wald tests were conducted. The results showed that the overall effect of baseline interocular SE difference on cure status was significant (Wald *χ*^2^ = 10.31, *p* = 0.016), while the nonlinear effect was not significant (Wald *χ*^2^ = 2.31, *p* = 0.315). However, both the overall effects of baseline interocular BCVA difference and baseline age on cure status were significant (Wald *χ*^2^ = 146.61, *p* < 0.001; Wald *χ*^2^ = 227.12, *p* < 0.001), and their nonlinear effects were also significant (Wald *χ*^2^ = 10.05, *p* = 0.007; Wald *χ*^2^ = 12.97, *p* = 0.002). Therefore, RCS was used for baseline interocular BCVA difference and baseline age to capture the nonlinear associations with the outcome, while baseline interocular SE difference was directly included as a linear term to establish Model 3. The AUC of Model 3 was 0.791 (95% CI 0.771, 0.811), with an AIC of 2160.91 and a BIC of 2205.87. The calibration curve indicated a Brier score of 0.177, but the Hosmer-Lemeshow test suggested poor model fit (Hosmer-Lemeshow *χ*^2^ = 16.07, *p* = 0.041). Ultimately, Regression Model 1 was still selected to construct the final nomogram prediction model. For this model, the AUC was 0.674 (95% CI 0.629, 0.718) in the population aged ≤7 years, and 0.775 (95% CI 0.750, 0.801) in the population aged >7 years.

**Figure 3:**
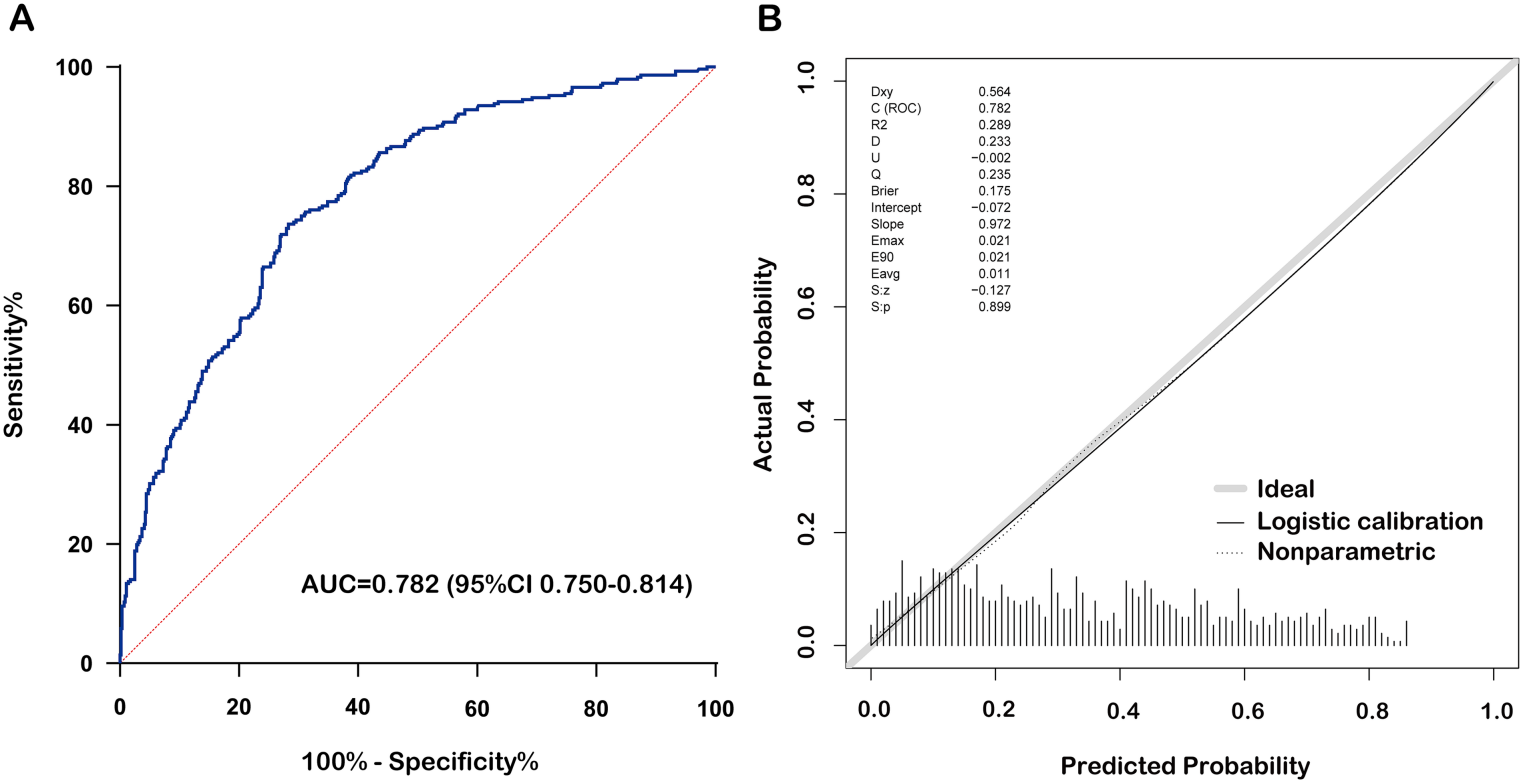
Performance of the nomogram prediction model in the validation set. **(A)** Receiver operating characteristic curve of nomogram for cure status prediction. **(B)** Calibration curve of nomogram for cure status prediction. BCVA, best corrected visual acuity.

**Figure 4:**
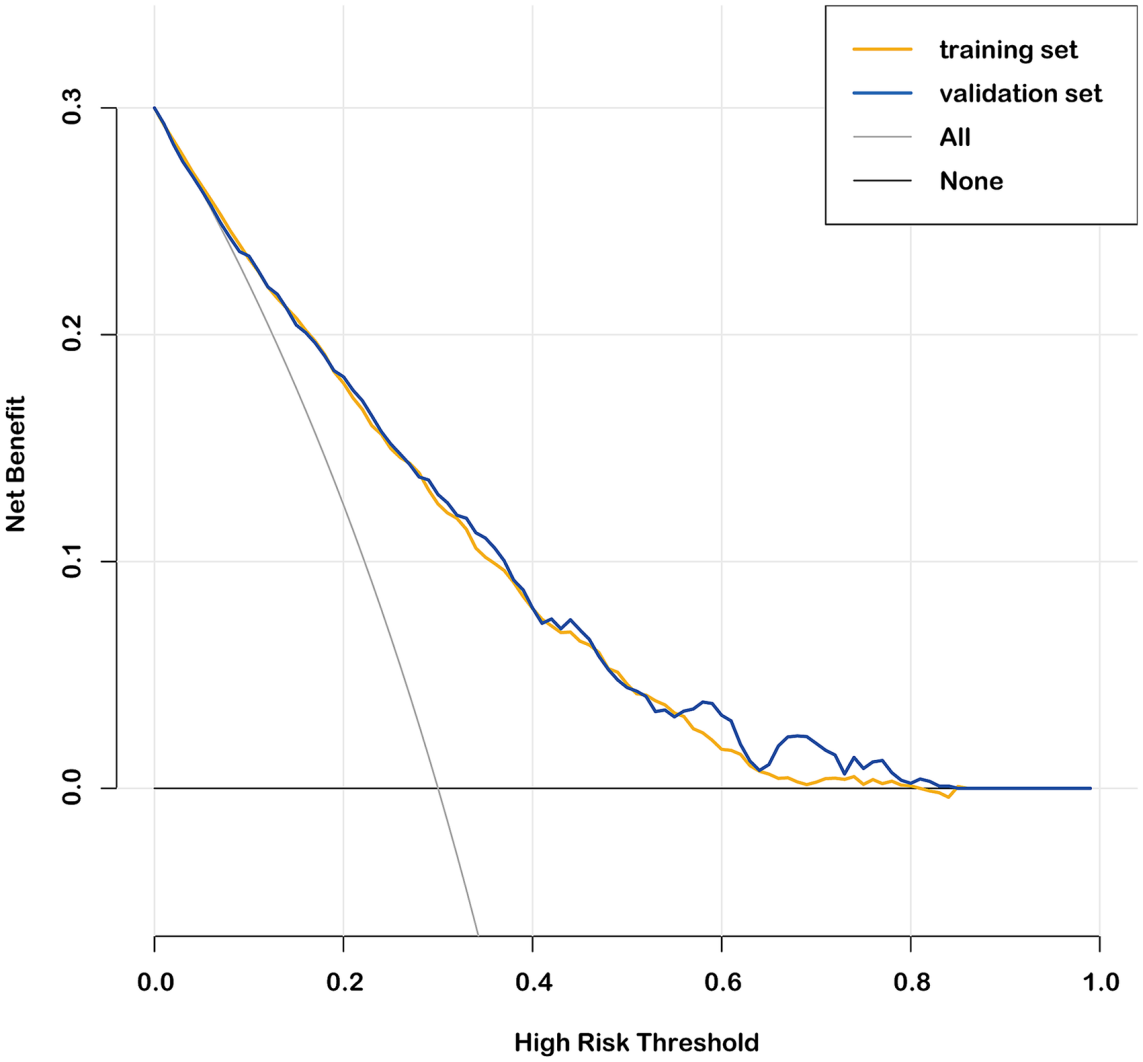
Decision curve analysis in the training set and validation set.

The nomogram prediction model estimates the probability of recovery from amblyopia after treatment by calculating a “total score,” which is the sum of the “scores” obtained for each parameter. The constructed nomogram prediction model was used to calculate the predicted probabilities in the validation set, and ROC curve analysis yielded an AUC of 0.782 (0.750–0.814) for the validation set (Figure 3A). The calibration curves for both the training and validation sets showed good agreement between the predicted and actual probabilities, with Brier scores of 0.179 for the training set (Figure 2D) and 0.175 for the validation set (Figure 3B). The DCA showed that the nomogram system achieved a higher net clinical benefit in both the training set and the validation set at most thresholds (0.1–0.8) (Figure 4).

## 4 Discussion

A retrospective analysis of 2,897 patients with anisometropic amblyopia was conducted, with a median follow-up of 2.83 years. The final age was 13.05 years, and the final BCVA of amblyopic eye was 0.22 LogMAR. During the treatment period, the difference in interocular BCVA improved from 0.52 LogMAR to 0.22 LogMAR. These findings align with those of the Pediatric Eye Disease Investigator Group (PEDIG) clinical trial, which reported a final visual acuity of 0.14 LogMAR and an interocular visual acuity difference of 0.21 LogMAR in 147 patients with amblyopia followed to age 15 (29). In this study, 35.2% of patients met the criteria for a cure, which is slightly lower than the 45.10% observed in a recent study of 102 patients with anisometropic amblyopia aged 4–14 years after 10.5 months (12). This difference may be attributed to the older age of the patients in the current study.

To the best of our knowledge, this study is the first to develop a nomogram-based prognostic model for anisometropic amblyopia, utilizing a large dataset (*n* = 2,897) and machine learning techniques for robust variable selection. Our findings align with prior research emphasizing age, interocular BCVA difference, and anisometropia severity as key prognostic indicators.

We found that age had the best discriminative ability for predicting the cure status of anisometropic amblyopia, with an AUC of 0.70 for the ROC curve and an OR of 0.790 in the final model. Previous research has consistently highlighted the importance of age in evaluating amblyopia treatment outcomes (8–11, 30–32). The PEDIG study found that treatment before the age of 5 years yielded more favorable outcomes (8). Holmes et al. also observed significantly poorer treatment effectiveness in children older than 7 years (9, 33). The reduced response to amblyopia treatment in older children may be related to a decrease in central nervous system plasticity and poorer compliance (9, 34). Therefore, early diagnosis and timely treatment are crucial.

The baseline difference in interocular BCVA was also an important factor in predicting prognosis, significantly affecting the cure status of anisometropic amblyopia, with an AUC of 0.6 for the ROC curve and an OR of 0.073 in the final model. This study found that patients with a smaller baseline difference in interocular BCVA had a higher probability of cure. Hong et al. found that in children aged 4–14 years with anisometropic amblyopia, those with a smaller baseline interocular BCVA difference were more likely to resolve amblyopia, indicating that better baseline BCVA in the amblyopic eye is associated with better treatment outcomes (12). In the MOTAS study, researchers found that the baseline BCVA in the amblyopic eye is a key predictor of treatment success. Patients with better initial BCVA in the amblyopic eye are more likely to achieve near-normal visual function after treatment (11). Additionally, their subsequent studies further highlighted that baseline BCVA in the amblyopic eye significantly impacts the treatment results of amblyopia (13).

A separate study on children aged 4–8 years with anisometropic amblyopia identified a significant positive correlation between anisometropia severity and final visual acuity (14). A study on visual changes 15 years after occlusion therapy for amblyopia further supports this finding (35). Similarly, Hong et al. found that unsuccessful amblyopia treatment was associated with a considerably larger interocular SE difference than successful cases. Specifically, the successful group showed an SE difference of 0.94 ± 2.71 D, while the unsuccessful group had a difference of 3.09 ± 3.05 D (12). These findings suggest that a greater degree of baseline anisometropia makes curing amblyopia more challenging. The present study also identified the baseline degree of anisometropia as a key factor affecting the cure status of patients with anisometropic amblyopia, with an AUC of 0.65 for the ROC curve and an OR of 0.903 in the final model. In general, an AUC (Area Under the Curve) above 0.60 for individual predictive factors in a nomogram is considered to indicate a certain level of predictive performance (36).

No studies have yet established a prognostic model using a nomogram to predict treatment outcomes for amblyopia, making it impossible to further evaluate the model performance in comparison with previous studies. However, some ophthalmological studies have already used nomograms to predict treatment outcomes. In research related to myopia, nomograms have been effectively applied to predict the treatment efficacy of orthokeratology in children with myopia or to assess the risk of myopia onset in school-aged children (21, 22, 24). The AUCs reported in these studies ranged from 0.77 to 0.87 for the training sets and 0.74 to 0.86 for the validation sets. Additionally, nomograms have been developed for predicting the likelihood of second surgeries in patients with concomitant esotropia (20). The AUCs reported in this study were 0.84 for the training set and 0.83 for the validation set. The nomogram prediction model constructed in this study achieved an AUC of 0.78 in both the training and validation sets, similar to those reported in previous studies, suggesting that this model has good potential for clinical application. In addition, The AUC values in the two age groups (≤7 years and >7 years) are relatively close, indicating that the prediction model has good generalizability.

Building upon these robust performance metrics, the nomogram demonstrates dual clinical utility by not only predicting treatment outcomes but also guiding clinical decision-making through data-driven strategies. For patients with low predicted cure rates, early intensive interventions, such as prolonged occlusion therapy or combined digital therapy, should be prioritized (37), while those with high predicted cure rates may benefit from minimal-intervention protocols. This tool enables precise resource allocation in high-demand pediatric ophthalmology services and reduces overtreatment burdens without compromising success rates. Furthermore, the nomogram could potentially enhance shared decision-making with parents by providing objective prognostic data, which might help improve treatment compliance (38). These features may contribute to improved amblyopia treatment efficacy.

This study has some limitations. First, because of its retrospective design based on actual clinical practice, indicators reflecting patient compliance could not be obtained. However, the relatively large sample size significantly mitigated the effect of non-compliant patients on the overall results. Second, because this study was retrospective and relied on data collected from the electronic medical record system, the available data were relatively limited. In future studies, incorporating additional clinical data, such as stereopsis, could further improve the predictive model.

## 5 Conclusion

Our study established a validated nomogram-based prediction model for anisometropic amblyopia prognosis. By incorporating age, interocular BCVA, and SE differences, this model can guide personalized treatment strategies in clinical settings. The nomogram provides a user-friendly scoring system for clinicians to estimate the probability of treatment success. This tool may contribute to personalized treatment planning, efficient resource allocation, and improved shared decision-making with parents, thus improving treatment efficacy.

## Data Availability

The raw data supporting the conclusions of this article will be made available by the authors, without undue reservation.
